# Safety and efficacy of low‐power pure‐cut hot snare polypectomy for small nonpedunculated colorectal polyps compared with conventional resection methods: A propensity score matching analysis

**DOI:** 10.1002/deo2.378

**Published:** 2024-05-07

**Authors:** Hidenori Kimura, Masayuki Oi, Kenichiro Imai, Takayuki Imai, Yukihiro Morita, Atsushi Nishida, Shigeki Bamba, Osamu Inatomi, Akira Andoh

**Affiliations:** ^1^ Department of Medicine Division of Digestive Endoscopy Shiga University of Medical Science Shiga Japan; ^2^ Department of Medicine Division of Gastroenterology Shiga University of Medical Science Shiga Japan; ^3^ Division of Gastroenterology Nagahama Red Cross Hospital Shiga Japan; ^4^ Division of Endoscopy Shizuoka Cancer Center Shizuoka Japan

**Keywords:** cold snare polypectomy, endoscopic mucosal resection, hot snare polypectomy, low‐power pure‐cut current, small colorectal polyps

## Abstract

**Objectives:**

Cold snare polypectomy (CSP) is widely performed for small colorectal polyps. However, small colorectal polyps sometimes include high‐grade adenomas or carcinomas that require endoscopic resection with electrocautery. This study aimed to evaluate the efficacy and safety of a novel resection technique, hot snare polypectomy with low‐power pure‐cut current (LPPC‐HSP) for small colorectal polyps, compared with CSP and conventional endoscopic mucosal resection (EMR).

**Methods:**

Records of patients who underwent CSP, EMR, or LPPC‐HSP for nonpedunculated colorectal polyps less than 10 mm between April 2021 and March 2022 were retrospectively evaluated. We analyzed and compared the treatment outcomes of CSP and EMR with those of LPPC‐HSP using propensity score matching.

**Results:**

After propensity score matching of 396 pairs, an analysis of CSP and LPPC‐HSP indicated that LPPC‐HSP had a significantly higher R0 resection rate (84% vs. 68%; *p* < 0.01). Delayed bleeding was observed in only two cases treated with CSP before matching. Perforation was not observed with either treatment. After propensity score matching of 176 pairs, an analysis of EMR and LPPC‐HSP indicated that their en bloc and R0 resection rates were not significantly different (99.4% vs. 100%, *p* = 1.00; 79% vs. 81%, *p* = 0.79). Delayed bleeding and perforation were not observed with either treatment.

**Conclusions:**

The safety of LPPC‐HSP was comparable to that of CSP. The treatment outcomes of LPPC‐HSP were comparable to those of conventional EMR for small polyps. These results suggest that this technique is a safe and effective treatment for nonpedunculated polyps less than 10 mm.

## INTRODUCTION

The removal of adenomatous colorectal polyps detected during colonoscopy can reduce the incidence and mortality of colorectal cancer.^1^, ^2^ Sub‐centimeter colorectal polyps are often encountered during colonoscopy in daily practice.^3^ The European Society of Gastrointestinal Endoscopy recommends cold snare polypectomy (CSP) as the first‐line therapy for small nonpedunculated polyps because of its higher safety versus hot snare polypectomy (HSP).^4^, ^5^ However, CSP cannot completely remove the muscularis mucosa, suggesting that the cutting plane of CSP is superficial.^6^, ^7^, ^8^, ^9^ Small colorectal polyps sometimes include high‐grade adenomas or carcinomas.^5^, ^10^, ^11^ CSP is considered unsuitable for the resection of such lesions because some high‐grade adenomas and carcinomas can invade the muscularis mucosa or submucosa. Therefore, endoscopic resection procedures using electrocautery, including HSP and endoscopic mucosal resection (EMR), should be performed for these lesions.^12^

Two randomized controlled trials that compared HSP with EMR for small colorectal polyps^13^, ^14^ showed significantly higher R0 resection rates and lower immediate post‐polypectomy bleeding rates in the EMR group, although the complete resection rates were similar. These results suggest that submucosal injection can improve the R0 resection rate^15^ and reduce thermal damage to deep blood vessels.^16^ Although EMR can be preferable to HSP regarding treatment outcomes, applying submucosal injection for sub‐centimeter polyps with possible advanced histology is time‐consuming, and prolonged procedure time may cause patients discomfort. Furthermore, submucosal injection potentially includes the theoretical risk of tumor seeding into the deep layers of the colon and the risk of local peritonitis if the injection is performed too deeply.^17^ Contrarily, HSP without submucosal injection is a simple resection method. However, HSP can cause deep thermal damage,^18^ with non‐negligible delayed bleeding (1.3%) and perforation rate (0.02%).^19^ Therefore, the development of less invasive therapeutic options for small colorectal polyps is required.

We focused on the setting of the electrosurgical unit. Conventional HSP has mainly been performed in the blend cut mode,^20^ which consists of both cut and coagulation current. A previous study^21^ reported that thermal damage to the external longitudinal muscle in pig models was 0% for the pure‐cut mode, 13.4% for the blend‐cut mode, and 53.4% for the coagulation mode, suggesting the risk of deep thermal damage with the current set to coagulation mode and safety with it set to pure‐cut mode. Based on this information, the efficacy and safety of HSP with low‐power pure‐cut current (LPPC‐HSP) have recently been reported for 10–14 mm flat and sessile colorectal adenomas.^22^ These results suggest that LPPC‐HSP may contribute less deep thermal damage to the muscle layer and vessels, which results in a lower risk of perforation and bleeding than conventional HSP with the blended cut current. Thus, LPPC‐HSP could be a minimally invasive treatment alternative to EMR for hot resection of not only nonpedunculated polyps 10–14 mm but also those less than 10 mm. However, little is known about the safety and efficacy of LPPC‐HSP for the treatment of small colorectal polyps. Therefore, this study investigated the safety and efficacy of LPPC‐HSP for nonpedunculated small colorectal polyps compared with CSP and conventional EMR.

## METHODS

### Study design and patients

From April 2021 to March 2022, consecutive patients who underwent CSP, EMR, or LPPC‐HSP for nonpedunculated colorectal polyps less than 10 mm at a university hospital were enrolled in this retrospective, observational study. The exclusion criteria were as follows: pathologically non‐neoplastic polyps resected using CSP, EMR, or LPPC‐HSP; inflammatory bowel diseases; polyposis syndrome; and hematologic disorders with thrombocytopenia or coagulopathy.

This study was approved by the Institutional Review Board of Shiga University of Medical Science (institutional no. R2022‐021) on June 6, 2022. Informed consent for participation in this study was obtained through an opt‐out method.

### Endoscopic procedures and patient management

Endoscopic resection with electrocautery for nonpedunculated polyps less than 10 mm was performed if the following endoscopic findings of advanced histology were present^10^: surface redness; white spots; nonpolypoid growth; loss of lobulation; heterogeneity or irregular capillary pattern on narrow‐band imaging; or inability to diagnose a low‐grade adenoma with high confidence. CSP was performed only for lesions confidently diagnosed as low‐grade adenomas less than 10 mm using image‐enhanced endoscopy with magnification.^12^ EMR and LPPC‐HSP were selected based on the endoscopist's preference. All endoscopic procedures were performed by nine experienced endoscopists (certified by the Japan Gastroenterological Endoscopy Society, each having performed >1500 colonoscopies and >100 polypectomies) and 10 gastrointestinal fellows (each having performed ≤1500 colonoscopies and ≤100 polypectomies). Gastrointestinal fellows always performed polypectomies under the supervision of experienced endoscopists. All procedures were performed using a high‐definition colonoscope (PCF‐H290ZI [Olympus Medical Co.] or EC‐L600Z [Fujifilm Co.]) with a disposable black rubber distal attachment (MAJ‐1990; Olympus Medical Co.). A standard electrosurgical generator (VIO300D or VIO3; ERBE) was used.

Polyps were measured using a nontraumatic catheter with a 3‐mm tip (Olympus Medical Co.) or forceps or snares of known dimensions. The morphology was defined according to the Paris classification.^23^

**FIGURE 1:**
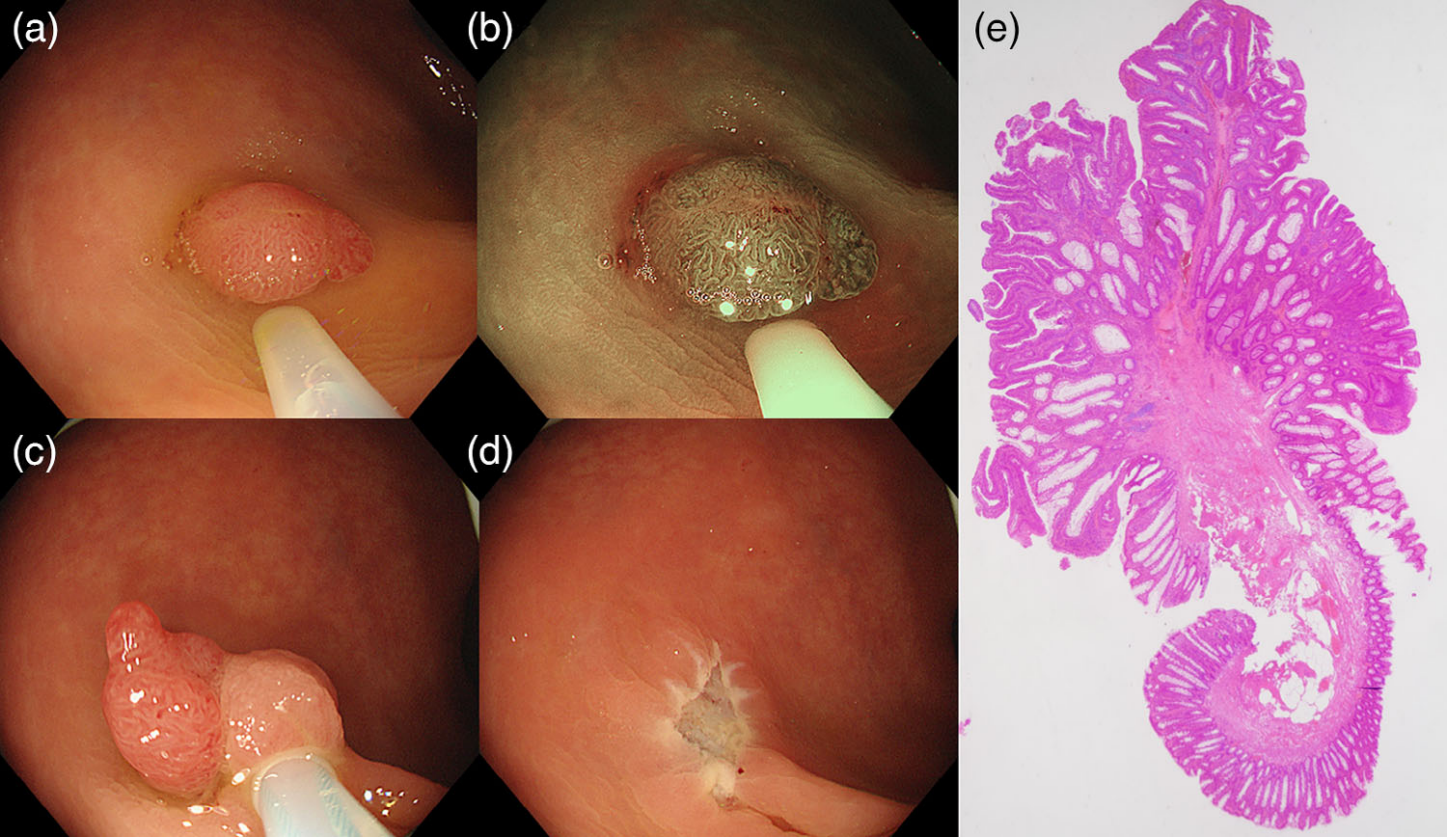
LPPC‐HSP procedure. (a) An 8‐mm sessile polyp located in the rectosigmoid colon. (b) The surface pattern observed with NBI is slightly different on the right side of the lesion, suggesting advanced histology. (c) The polyp is snared and resected with the low‐power pure‐cut current. (d) The ulcer after LPPC‐HSP. (e) Pathological examination reveals intramucosal cancer with tumor‐free horizontal and vertical margins. LPPC‐HSP, low‐power pure‐cut hot snare polypectomy; NBI, narrow‐band imaging.

For CSP, a 10‐ or 15‐mm oval snare with a wire diameter of 0.30 mm (SnareMaster Plus; Olympus Medical Co.) was used to snare and resect the polyp without the use of electrocautery and submucosal injection. For EMR, saline solution was injected into the submucosal layer. Then, using the 10‐ or 15‐mm oval snare, the polyp was closely snared and resected with the blend‐cut current (ENDOCUT Q; effect, 3; time interval, 2; time duration, 2). For LPPC‐HSP, using the oval snare without submucosal injection, the polyp was snared and resected with a low‐power pure‐cut current (Autocut; VIO300D: effect, 1 [10 W]; VIO3: effect, 0.4 [Pmax 13 W]; Figure 1 and Video S1). Prophylactic clipping was performed based on the endoscopist's preference.

The resected specimens were retrieved using button‐attached suction, forceps, or nets. Specimens were fixed in 10% formalin and sent to the pathology department for histological assessment. The histological diagnosis was made by experienced pathologists according to the Japanese Classification of Colorectal Carcinoma.^24^

In daily practice at our hospital, patients treated with CSP were followed up as outpatients, and patients treated with EMR or LPPC‐HSP were briefly admitted as inpatients. The patients were followed up as outpatients for 2–4 weeks after colonoscopy.

### Data collection and definition

Clinicopathological information was collected retrospectively from the institutional electronic medical records. We analyzed the short‐term treatment outcomes, including the en bloc resection rate, R0 resection rate, and adverse events such as immediate or delayed bleeding and perforation. The main outcomes were the adverse event rate in safety analysis between CSP and LPPC‐HSP, and the R0 resection rate in efficacy analysis between EMR and LPPC‐HSP.

En bloc resection was defined as the removal of the polyp in one piece, without visible neoplasm at the ulcer margins. R0 resection was defined as en bloc resection with tumor‐free margins confirmed by a histological examination. If the specimen was fragmented including the neoplastic area, it was considered as RX resection. Immediate bleeding was defined as intraprocedural bleeding that required hemostasis (such as spurting bleeding or continuous oozing that did not stop spontaneously within 30 seconds). Delayed bleeding was defined as postprocedural bleeding that required hospitalization or repeat colonoscopy. If the bleeding point could be identified, only that lesion was considered to have delayed bleeding; if bleeding occurred but the source of the bleeding could not be identified, all lesions in the patient resected on that day were considered to have delayed bleeding. Perforation was defined as a defect in the muscular layer of a CSP/EMR/LPPC‐HSP ulcer.

### Statistical analysis

All continuous variables are reported as the median and interquartile range. All categorical variables are reported as the number (percentage). We used the Mann–Whitney U test to compare continuous variables. Fisher's exact test was performed for categorical variables. Statistical significance was set at *p* < 0.05. We performed propensity score matching to reduce selection bias for each lesion included in the CSP and LPPC‐HSP groups and each lesion included in the EMR and LPPC‐HSP groups. We used a ratio of 1:1 and nearest‐neighbor matching without replacement within a caliper width of 0.2 of the standard deviation for the logit of the propensity score. The following variables expected to affect treatment outcomes were selected: age; sex; polyp size; morphology; lesion location (right colon, left colon, or rectum); endoscopist (experienced endoscopist or gastrointestinal fellow); and antithrombotic agents. Standardized differences were used to diagnose the baseline balance after matching. A standardized difference of less than 0.1 represented an adequate variable balance between groups. The propensity score was calculated using a logistic regression model. The adequacy of the propensity score matching models was evaluated with c‐statistics in receiver operating characteristic analysis. After matching, the clinical outcomes of the matched pairs were compared. All statistical analyses were conducted using EZR software version 1.55 (Saitama Medical Center, Jichi Medical University).

## RESULTS

### Patients and lesions

**FIGURE 2:**
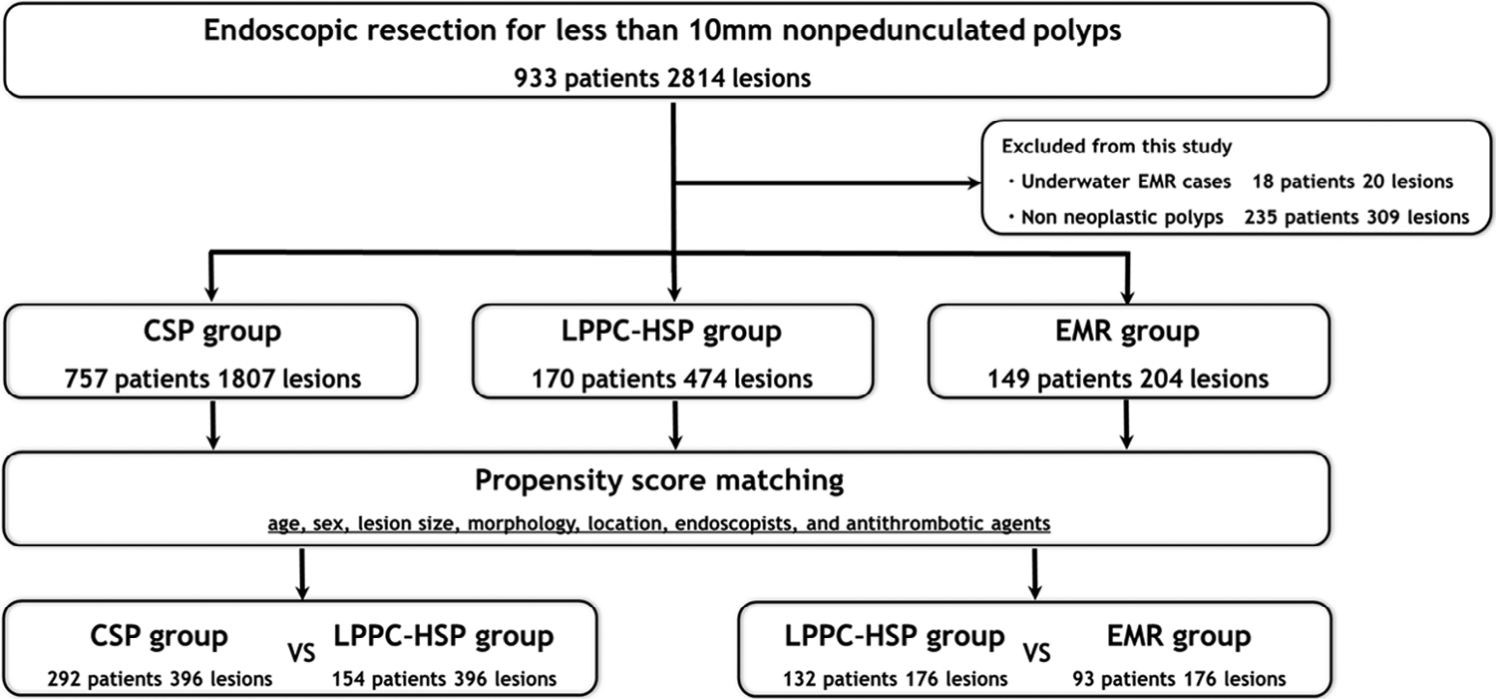
Patient flow diagram. CSP, cold snare polypectomy; EMR, endoscopic mucosal resection; LPPC‐HSP, low‐power pure‐cut hot snare polypectomy.

A total of 933 patients (2814 lesions) underwent endoscopic resection for non‐pedunculated colorectal polyps less than 10 mm at our institution. Finally, 1807 CSP cases (757 patients and 1807 lesions), 204 EMR cases (149 patients and 204 lesions), and 474 LPPC‐HSP cases (170 patients and 474 lesions) were included in this study. A patient flow diagram is shown in Figure 2.

### Analysis of treatment outcomes of CSP and LPPC‐HSP

The baseline characteristics of the patients are summarized in Table 1. The sessile morphology proportion and lesion size of the LPPC‐HSP group were significantly larger than those of the CSP group (35% vs. 16% [*p* < 0.01]; 5 mm vs. 4 mm [*p* < 0.01]). The c‐statistic of the matching model was 0.764. After propensity score matching of 396 pairs, the baseline characteristics of the groups were comparable.

**TABLE 1:** Baseline characteristics of the cold snare polypectomy (CSP) and low‐power pure‐cut hot snare polypectomy (LPPC‐HSP) groups.

	Before propensity score matching			After propensity score matching			
	CSP	LPPC‐HSP		CSP	LPPC‐HSP		Standardized
Lesions	*n* = 1807	*n* = 474	*p‐*value	*n* = 396	*n* = 396	*p‐*value	difference
Age, years (IQR)	72 (66–78)	72 (67–77)	0.94	73 (67–78)	72 (67–77)	0.11	0.09
Male sex (%)	1257 (70)	314 (66)	0.18	267 (67)	274 (69)	0.65	0.04
Lesion size, mm (IQR)	4 (3–5)	5 (4–7)	<0.01	5 (4–6)	5 (4–6)	0.80	<0.01
Location (%)			0.06			0.81	0.04
Right‐side colon	1254 (69)	324 (68)		260 (66)	268 (68)		
Left‐side colon	479 (27)	140 (30)		126 (32)	118 (30)		
Rectum	74 (4.1)	10 (2.1)		10 (2.5)	10 (2.5)		
Morphology (%)			<0.01			0.31	0.08
0‐Is	286 (16)	166 (35)		105 (27)	119 (30)		
0‐IIa	1521 (84)	307 (65)		291 (74)	277 (70)		
0‐IIc	0 (0)	1 (0.2)		0 (0)	0 (0)		
Antithrombotic agents (%)	388 (22)	89 (19)	0.21	79 (20)	77 (19)	0.93	0.01
Gastrointestinal fellows performed (%)	1223 (68)	364 (77)	<0.01	297 (75)	298 (75)	1.00	<0.01

Standardized difference <0.1 represents adequate variable balance between groups.

Abbreviations: CSP, cold snare polypectomy; LPPC‐HSP, hot snare polypectomy with low‐power pure‐cut current; IQR, interquartile range.

The treatment outcomes of CSP and LPPC‐HSP are shown in Table 2. After propensity score matching, en bloc resection was achieved for all cases in both groups, but the R0 resection rate of the LPPC‐HSP group was significantly higher than that of the CSP group (84% vs. 68%; *p* < 0.01). Regarding adverse events, delayed bleeding was observed in only two CSP cases (a patient with chronic renal failure taking warfarin and a patient taking aspirin) before matching. Perforation was not observed in either of these groups.

**TABLE 2:** Treatment outcomes of the cold snare polypectomy (CSP) and low‐power pure‐cut hot snare polypectomy (LPPC‐HSP) groups.

	Before propensity score matching			After propensity score matching		
	CSP	LPPC‐HSP		CSP	LPPC‐HSP	
Lesions	*n* = 1807	*n* = 474	*p‐*value	*n* = 396	*n* = 396	*p‐*value
En bloc resection, *n* (%)	1806 (99.9)	474 (100)	1.00	396 (100)	396 (100)	N/A
R0 resection, *n* (%)	1351 (75)	390 (82)	<0.01	270 (68)	332 (84)	<0.01
Pathology, *n* (%)			<0.01			0.06
Low‐grade adenoma	1638 (91)	388 (82)		356 (90)	331 (84)	
High‐grade adenoma	17 (0.9)	8 (1.7)		5 (1.3)	6 (1.5)	
Intramucosal cancer	0 (0)	1 (0.2)		0 (0)	1 (0.3)	
Sessile serrated lesion	143 (7.9)	69 (15)		34 (8.6)	52 (13)	
Sessile serrated lesion with dysplasia	8 (0.4)	5 (1.1)		1 (0.3)	3 (0.8)	
Traditional serrated adenoma	1 (0.1)	3 (0.6)		0 (0)	3 (0.8)	
Prophylactic clipping after resection, *n* (%)	10 (0.6)	26 (5.5)	<0.01	1 (0.3)	20 (5.1)	<0.01
Immediate bleeding, *n* (%)	15 (0.8)	2 (0.4)	0.55	1 (0.3)	2 (0.5)	1.00
Delayed bleeding, *n* (%)	2 (0.1)	0 (0)	1.00	0 (0)	0 (0)	N/A
Perforation, *n* (%)	0 (0)	0 (0)	N/A	0 (0)	0 (0)	N/A

Abbreviations: CSP, cold snare polypectomy; LPPC‐HSP, hot snare polypectomy with low‐power pure‐cut current; IQR, interquartile range; N/A, not available.

### Analysis of treatment outcomes of EMR and LPPC‐HSP

The baseline characteristics of the patients are summarized in Table 3. The sessile morphology proportion and lesion size of the LPPC‐HSP group were significantly smaller than those of the EMR group (35% vs. 64% [*p* < 0.01]; 5 mm vs. 7 mm [*p* < 0.01]). Endoscopic procedures for cases in both groups were mainly performed by gastrointestinal fellows (89% [EMR] vs. 77% [LPPC‐HSP]; *p* < 0.01). The c‐statistic of the matching model was 0.759. After the propensity score matching of 176 pairs, the baseline characteristics of the groups were comparable.

**TABLE 3:** Baseline characteristics of the endoscopic mucosal resection and low‐power pure‐cut hot snare polypectomy groups.

	Before propensity score matching			After propensity score matching			
	EMR	LPPC‐HSP		EMR	LPPC‐HSP		Standardized
Lesions	*n* = 204	*n* = 474	*p‐*Value	*n* = 176	*n* = 176	*p‐*Value	difference
Age, years (IQR)	72 (64–76)	72 (67–77)	0.04	72 (65–77)	70 (67–77)	0.83	0.03
Male sex (%)	149 (73)	314 (66)	0.09	124 (71)	124 (71)	1.00	<0.01
Lesion size, mm (IQR)	7 (6–8)	5 (4–7)	<0.01	6 (5–8)	6 (5–8)	0.91	0.02
Location (%)			<0.01			0.75	0.08
Right‐side colon	130 (64)	324 (68)		112 (64)	119 (68)		
Left‐side colon	59 (29)	140 (30)		55 (31)	49 (28)		
Rectum	15 (7.4)	10 (2.1)		9 (5.1)	8 (4.5)		
Morphology (%)			<0.01			1.00	<0.01
0‐Is	131 (64)	166 (35)		104 (59)	104 (59)		
0‐IIa	72 (35)	307 (65)		72 (41)	72 (41)		
0‐IIc	1 (0.5)	1 (0.2)		0 (0)	0 (0)		
Antithrombotic agents (%)	27 (13)	89 (19)	0.10	26 (15)	30 (17)	0.66	0.06
Gastrointestinal fellows performed (%)	182 (89)	364 (77)	<0.01	155 (88)	162 (92)	0.29	0.13

Standardized difference <0.1 represents adequate variable balance between groups.

Abbreviations: EMR, endoscopic mucosal resection; LPPC‐HSP, hot snare polypectomy with low‐power pure‐cut current; IQR, interquartile range.

The treatment outcomes of EMR and LPPC‐HSP are shown in Table 4. LPPC‐HSP was not converted to EMR for any cases. After propensity score matching, the en bloc and R0 resection rates of the EMR and LPPC‐HSP groups were not significantly different (99.4% vs. 100% [*p* = 1.00]; 79% vs. 81% [*p* = 0.79]). Submucosal invasive cancer was detected in one case in the EMR group, and intramucosal cancer was detected in two cases in the EMR group and in one case in the HSP group. All these cases were resected with tumor‐free margins.

**TABLE 4:** Treatment outcomes of the endoscopic mucosal resection (EMR) and low‐power pure‐cut hot snare polypectomy (LPPC‐HSP) groups.

	Before propensity score matching			After propensity score matching		
	EMR	LPPC‐HSP		EMR	LPPC‐HSP	
Lesions	*n* = 204	*n* = 474	*p*‐value	*n* = 176	*n* = 176	*p*‐value
En bloc resection, *n* (%)	202 (99)	474 (100)	0.09	175 (99.4)	176 (100)	1.00
R0 resection, *n* (%)	162 (79)	390 (82)	0.39	139 (79)	142 (81)	0.79
Pathology, *n* (%)			<0.01			0.19
Low‐grade adenoma	164 (80)	388 (82)		140 (80)	140 (80)	
High‐grade adenoma	16 (7.8)	8 (1.7)		12 (6.8)	7 (4.0)	
Intramucosal cancer	2 (1.0)	1 (0.2)		2 (1.1)	1 (0.6)	
Superficial submucosal invasive cancer	1 (0.5)	0 (0)		1 (0.6)	0 (0)	
Sessile serrated lesion	17 (8.3)	69 (15)		17 (9.7)	27 (15)	
Sessile serrated lesion with dysplasia	1 (0.5)	5 (1.1)		1 (0.6)	1 (0.6)	
Traditional serrated adenoma	3 (1.5)	3 (0.6)		3 (1.7)	0 (0)	
Prophylactic clipping after resection, *n* (%)	74 (36)	26 (5.5)	<0.01	63 (36)	11 (6.2)	<0.01
Immediate bleeding, *n* (%)	9 (4.4)	2 (0.4)	<0.01	8 (4.5)	0 (0)	<0.01
Delayed bleeding, *n* (%)	0 (0)	0 (0)	N/A	0 (0)	0 (0)	N/A
Perforation, *n* (%)	0 (0)	0 (0)	N/A	0 (0)	0 (0)	N/A

Abbreviations: EMR, endoscopic mucosal resection; LPPC‐HSP, hot snare polypectomy with low‐power pure‐cut current; N/A, not available.

Regarding adverse events, immediate bleeding was observed in 4.5% of cases in the EMR group and 0% of cases in the LPPC‐HSP group (*p* < 0.01). Prophylactic clipping was performed for 36% (63/176) of cases in the EMR group and 6.2% (11/176) of cases in the LPPC‐HSP group (*p* < 0.01). Delayed bleeding and perforation were not observed in either of these groups.

## DISCUSSION

Herein, we demonstrated that the safety of LPPC‐HSP was comparable to that of CSP. Furthermore, we demonstrated that the treatment outcomes, including en bloc and R0 resection rates, of LPPC‐HSP were comparable to those of conventional EMR for small polyps. These results suggest that LPPC‐HSP may be one of the minimally invasive treatments for hot resection of small colorectal tumors.

The main finding of our study was that LPPC‐HSP was not inferior to conventional EMR in terms of en bloc resection and R0 resection. A previous study reported a lower R0 resection rate of conventional HSP for 5–10 mm colorectal polyps than that of EMR (49.7% vs. 74.8%),^14^ suggesting that submucosal injection can improve the R0 resection rate. The blend‐cut mode includes not only the cut current but also the coagulation current, which may have caused thermal damage to the specimen without submucosal injection, resulting in unevaluable tumor margins. However, LPPC‐HSP was performed using a pure‐cut current without a coagulation component, which may have resulted in less thermal damage to the specimens and, thus, the R0 resection rate comparable to that of EMR. Therefore, for small polyps with possible advanced histology that require endoscopic resection with electrocautery and a detailed pathological evaluation, LPPC‐HSP might be a more convenient treatment with resection ability comparable to that of EMR.

The advantage of LPPC‐HSP is its simplicity. LPPC‐HSP does not require submucosal injection and comprises a short procedure time (similar to CSP); furthermore, the resection ability of LPPC‐HSP is similar to that of EMR. A previous study^5^ reported that the procedure time of HSP without submucosal injection was similar to that of CSP (65 s vs. 60 s), whereas the procedure time of EMR was nearly twice as long (116 s). The procedure time of underwater EMR is almost the same as that of EMR.^25^ These results suggest that among endoscopic resection methods with electrocautery, HSP has the shortest procedure time. Additionally, because LPPC‐HSP might cause less deep thermal damage, resulting in lower risks of perforation and bleeding, prophylactic clipping may not be required after resection. During this study, prophylactic clipping was performed for 36% of cases in the EMR group but only 6.2% of cases in the LPPC‐HSP group. Nevertheless, the number of adverse events did not increase in the LPPC‐HSP group. Therefore, we consider it important to adopt LPPC‐HSP for hot resection of small polyps, not only because of the shorter procedure time but also because of the reduced burden of performing prophylactic measures after resection.

Compared with CSP, LPPC‐HSP did not result in a significant increase in adverse events; delayed bleeding was observed in only two cases treated with CSP. Compared with HSP, CSP involves shallower resection depths^8^ and less damage to the submucosal arteries; this difference in the resection depth may be related to the frequency of adverse events.^26^ Additionally, the resection depth of LPPC‐HSP is shallower than that of conventional HSP using the blend‐cut current,^22^ which may cause less damage to the arteries in the submucosal layer, leading to fewer adverse events than those associated with conventional resection with electrocautery and safety comparable to that of CSP. Although this study suggested the safety of LPPC‐HSP, the sample size was insufficient to evaluate delayed adverse events. To evaluate the safety profile of LPPC HSP, a multicenter prospective trial with a large sample size is needed.

This study has some limitations. First, it was a retrospective study performed at a single medical center, thus resulting in potential selection bias. Further multicenter studies are required to validate our results. Second, most lesions were removed by gastrointestinal fellows, thus limiting the generalizability of the results. However, because LPPC‐HSP resulted in treatment outcomes comparable to those of EMR, even when performed by gastrointestinal fellows, similar or better treatment outcomes can be expected in general clinical practice. Third, the prevalence of small polyps with advanced histology in this study was low and the evaluation of resection outcomes for such lesions is somewhat insufficient. During a prospective study of LPPC‐HSP,^22^ the containing muscularis mucosa rate was 100% and the submucosal tissue rate was 89%, which was comparable to that of conventional HSP (81%)^8^ and EMR (92%)^6^ for small polyps. Additionally, intramucosal cancer can be removed with tumor‐free horizontal and vertical margins using LPPC‐HSP (Figure 1). LPPC‐HSP is expected to be as capable of resecting submucosal tissue as conventional HSP and EMR. Although LPPC‐HSP should not be applied for resection of submucosal invasive cancer due to the shallower submucosal resection depth than conventional HSP^22^, LPPC‐HSP, which can completely resect at least to the muscularis mucosa might be a minimally invasive treatment alternative to EMR for nonpedunculated polyps less than 10 mm with possible advanced histology up to intramucosal cancer. Further investigation is required regarding the resection ability and usefulness of LPPC‐HSP for such lesions.

In conclusion, the safety of LPPC‐HSP was comparable to that of CSP. Furthermore, the treatment outcomes of LPPC‐HSP were comparable to those of conventional EMR for small polyps. These results suggest that this technique is a safe and effective treatment for nonpedunculated polyps less than 10 mm.

## CONFLICT OF INTEREST STATEMENT

None.

## Supporting information


**VIDEO S1** An 8‐mm sessile polyp is located in the rectosigmoid colon. The surface pattern observed with narrow‐band imaging is slightly different on the right side of the lesion, suggesting advanced histology. The polyp is snared and resected with the low‐power pure‐cut current. Pathological examination reveals intramucosal cancer with tumor‐free horizontal and vertical margins.
